# Scanning Electrochemical Impedance Microscopy-Based Assessment of Glucose Biosensors

**DOI:** 10.1021/acs.langmuir.5c02292

**Published:** 2025-08-04

**Authors:** Antanas Zinovicius, Timas Merkelis, Juste Rozene, Sigita Bendinskaite, Inga Morkvenaite, Sheng-Tung Huang, Arunas Ramanavicius

**Affiliations:** † Department of Mechatronics, Robotics, and Digital Manufacturing, 112678 Vilnius Gediminas Technical University, Plytinės g. 25, 10105 Vilnius, Lithuania; ‡ Department of Nanotechnology, Center for Physical Sciences and Technology, Saulėtekio 3, 10257 Vilnius, Lithuania; § Department of Physical Chemistry, 54694 Vilnius University, Naugarduko 24, 03225 Vilnius, Lithuania; ∥ Department of Electronics Engineering, Vilnius Gediminas Technical University, Plytinės g. 25, 10105 Vilnius, Lithuania; ⊥ Department of Chemical Engineering and Biotechnology and Institute of Biochemical and Biomedical Engineering, 34877 National Taipei University of Technology, 106 Taipei, Taiwan; # High-Value Biomaterials Research and Commercialization Center, National Taipei University of Technology, 10608 Taipei, Taiwan

## Abstract

Scanning electrochemical impedance microscopy (SEIM) was assessed as an electrochemical method for developing glucose biosensors based on glucose oxidase (GOx). To determine the lowest detectable GOx activity, scanning electrochemical microscopy (SECM) in the feedback mode (FB-SECM) was applied. During the measurement procedure, an ultramicroelectrode (UME) was moved vertically over the surface modified by immobilized GOx. A positive feedback response of the FB-SECM mode was determined during the assessment of surfaces modified by 5 fg/mm^2^ to 20 μg/mm^2^ surface concentration of GOx. The lowest surface concentration of GOx, which still provided reliable measurement results, was 50 fg/mm^2^. The approach curves registered using the FB-SECM mode were assessed using a mathematical model adapted for the calculation of reaction kinetics by SECM. According to this model, the reaction kinetics constant λ was calculated for differently modified surfaces in the presence of the same glucose concentration. For the surface not modified by GOx, the constant λ was determined to be 0.14, while for the GOx-modified surface λ gradually increased with increasing GOx surface concentrations, the λ value reached 0.34 when it was determined on the surfaces modified by 500 ng/mm^2^ of GOx. Any statistically significant changes in FB-SECM were detected when the surface concentration of GOx exceeded 50 pg/mm^2^. Notably, localized electrochemical impedance measurements using the SEIM mode enabled one to detect GOx activity even when GOx was immobilized on a nonconductive substrate surface. The results show that redox competition-based SEIM can be used to determine glucose concentrations in the range of 2–20 mM, while using 10 Hz AC perturbation. An FB-SECM configuration allows the reuse of the ultramicroelectrode while providing the localized impedance-based glucose concentration and enzyme activity measurements.

## Graphical abstract

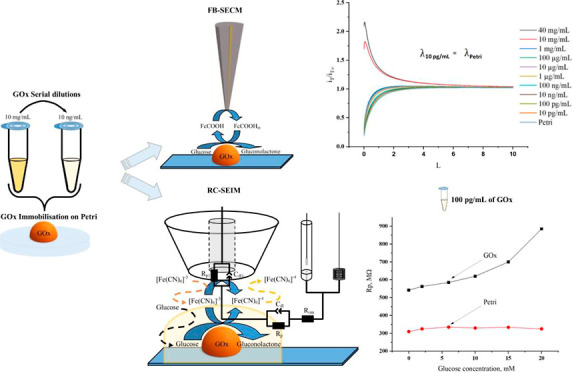

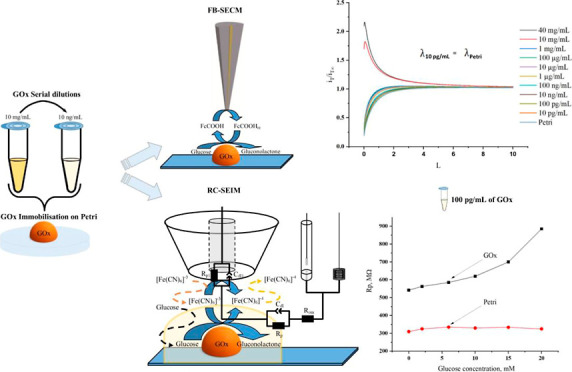

## Introduction

Over the past decade, the demand for glucose sensors has significantly increased. These glucose sensors can be based on various detection methods: optical, mass, or electrochemical signals.^1^ The analyte detection techniques for optical glucose biosensors are primarily based on the determination of surface plasmon resonance, photoluminescence, and spectrophotometric signals. These methods often require expensive equipment, consume significant energy, and are sensitive to colored and/or optically active materials in blood samples.^2–4^ In molecular imprint technology, the sensing is limited to a narrow concentration range. It requires a quartz crystal microbalance, which is still relatively expensive and sensitive to environmental conditions, namely temperature.^5^ Electrochemical glucose biosensors mostly rely on amperometric measurements, which offer rapid analysis, the ability for continuous monitoring, and simple maintenance.^6,7^ In electrochemical biosensors, enzymes are the most frequently applied as biorecognition elements. Glucose oxidase (GOx) is one of the most popular choices as it is readily available, shows high catalytic activity, sensitivity, selectivity, resilience to unfavorable pH, ionic strength, and temperature conditions.^8^ However, electrochemical glucose biosensors also have several limitations, including some difficulties related to enzyme immobilization on the electrode, thermal, and chemical instability of enzymes, limited sensor sensitivity, and a limited sensor lifespan due to enzyme desorption or denaturation, which can lead to impaired performance after extended use.^9,10^ Signal detection in electrochemical biosensors can be performed by various electrochemical methods, including amperometry, potentiometry, coulometry, and electrochemical impedance spectroscopy (EIS).^11,12^

Developing cost-effective glucose biosensors is crucial for their widespread adaptation in various applications. Glucose oxidase (GOx) has been extensively studied as a sensing element in these biosensors for over half a century. Efforts to increase the analytical signal have been focused on incorporating nanomaterials such as carbon nanotubes, metal nanoparticles, and redox conductive polymers, as well as designing nanocomposites using a combination of these materials.^13^ However, the quantity of GOx immobilized on surfaces can vary significantly, ranging from 1 to 100 mg/mL, having one of the highest contributions toward cost per sensing unit.^14–18^ Various immobilization methods have been explored, including the direct deposition of the enzyme solution on an electrode, soaking and drying, and more complex techniques involving reagents that form covalent bonds with the enzyme and/or substrate used for immobilization.^14–18^ To address the challenge of the cost associated with the enzyme usage, we focused on minimizing the utilization of enzymes in GOx-based biosensors while maintaining their analytical performance.

Scanning electrochemical microscopy (SECM) is a technique that allows one to perform electrochemical measurements over a sample surface and determine local electrochemical activity without invasive procedures, thus allowing the same sample to be assessed multiple times while performing measurements in the desired medium.^19^ SECM enables the evaluation of redox enzymes immobilized on conductive and insulating surfaces because the measuring electrode is an ultramicroelectrode (UME).

To determine the reaction kinetics of the redox enzyme-modified surface, data from approach curves registered by scanning electrochemical microscopy in the feedback mode (FB-SECM) can be evaluated by fitting the corresponding mathematical models.^20^ In the FB-SECM mode, current is directly proportional to the concentration of the redox species and this could pose a potential limitation of this mode when measuring low concentrations of these species.^21^ To resolve this issue and enhance signals, a redox competition (RC-SECM) mode was introduced. In the RC-SECM mode, both UME and the substrate consume the same redox active species, albeit undergoing different redox reactions within the electrochemical system. In the RC-SECM mode, due to the consumption of redox active species, the measured current in the narrow gap between the electrode and the enzyme-modified surface decreases.^22,23^ The RC-SECM mode offers advantages beyond its higher-resolution imaging capabilities. The UME and substrate consume the same reactant, allowing a competition-based approach and providing valuable insights into localized reactivity and species distribution.^24^ However, potential interferences arising from overlapping redox potentials or competing reactions can complicate the interpretation of signals in the RC-SECM mode.^25^ Despite these challenges, the RC-SECM mode is a powerful tool for imaging electrochemical activity and localized reactivity.

Alternating current SECM (AC-SECM) involves scanning the sample by applying one specific voltage frequency.^26^ Compared with direct current modes, AC-SECM has improved signal-to-noise ratios, making it particularly useful for probing weak electrochemical processes and localized events in complex samples. When the electrochemical impedance spectra (EIS) are registered using SECM, they are called scanning electrochemical impedance microscopy (SEIM). Using this technique, it is possible to obtain information about localized redox activity dependence at many applied frequencies and then analyze data by fitting mathematical models. They are based on equivalent electrical circuits and provide information about charge transfer, double-layer capacitance, and diffusion in the electrode/electrolyte interface.^15,27,28^ In previous works, we successfully detected areas covered by antibodies conjugated with horseradish peroxidase using the RC-SEIM mode.^27^ RC-SEIM was applied to detect a wide range of hydrogen peroxide concentrations with a small amount of enzymes. GOx at low concentrations was used as an electrochemical label in a sandwich format immunoassay, detected by FB-SECM.^29^

In this study, a novel biosensing approach that applies SECM in the SEIM-mode, which enables glucose determination on substrates modified by a low GOx surface concentration, was developed. Unlike conventional electrochemical biosensors, where the biorecognition element is directly immobilized on the working electrode, the proposed design immobilizes GOx on a nonconductive disposable Petri dish, while keeping the UME unmodified and, therefore, reusable for an unlimited number of glucose determinations. Such a modular SEIM-based biosensor architecture significantly reduces fabrication costs, enables a practically unlimited reuse of the working electrode, and allows for the fast replacement of the substrate modified by immobilized GOx. It was demonstrated that glucose concentrations can be determined by both FB-SECM and RC-SEIM modes performed over the GOx-modified surface. This eliminates the need for a bulk signal interpretation and opens new possibilities for the development of biosensing systems.

## Results and Discussion

### Experiments in the FB-SECM Mode

Immobilization of different GOx surface concentrations (ranging from 5 fg/mm^2^ to 20 μg/mm^2^) was performed by a glutaraldehyde-based cross-linking procedure. The GOx-modified surface activity measurements were performed in the FB-SECM mode while moving UME vertically toward the sample surface. Figure 1A shows that the approach curves showed a typical positive feedback behavior when the surface was modified by 5 μg/mm^2^ and 20 μg/mm^2^ of the GOx enzyme. By further decreasing GOx concentrations, the approach curve was expected to continue to show a positive feedback behavior. However, experimental data showed behavior similar to a negative feedback (current decreased approaching GOx-modified surface), indicating lower catalytic activity than expected. Experimental data were fitted to a mathematical model^30,31^ from which the kinetic constant λ was calculated and was assessed as a value characterizing analytical signal. The lowest detectable GOx concentration (Figure 1B) was 5 fg/mm^2^ when the kinetic constant (λ) reached 0.16, and decreasing the concentration further to 5 fg/mm^2^ (orange dashed line) kinetic constant as expected decreased and reached 0.14, which is the same as the λ of the nonmodified surface (purple line). This decrease shows that catalytic activity near the surface covered by 5 fg/mm^2^ GOx is the same as that on an unmodified surface.

**Figure 1:**
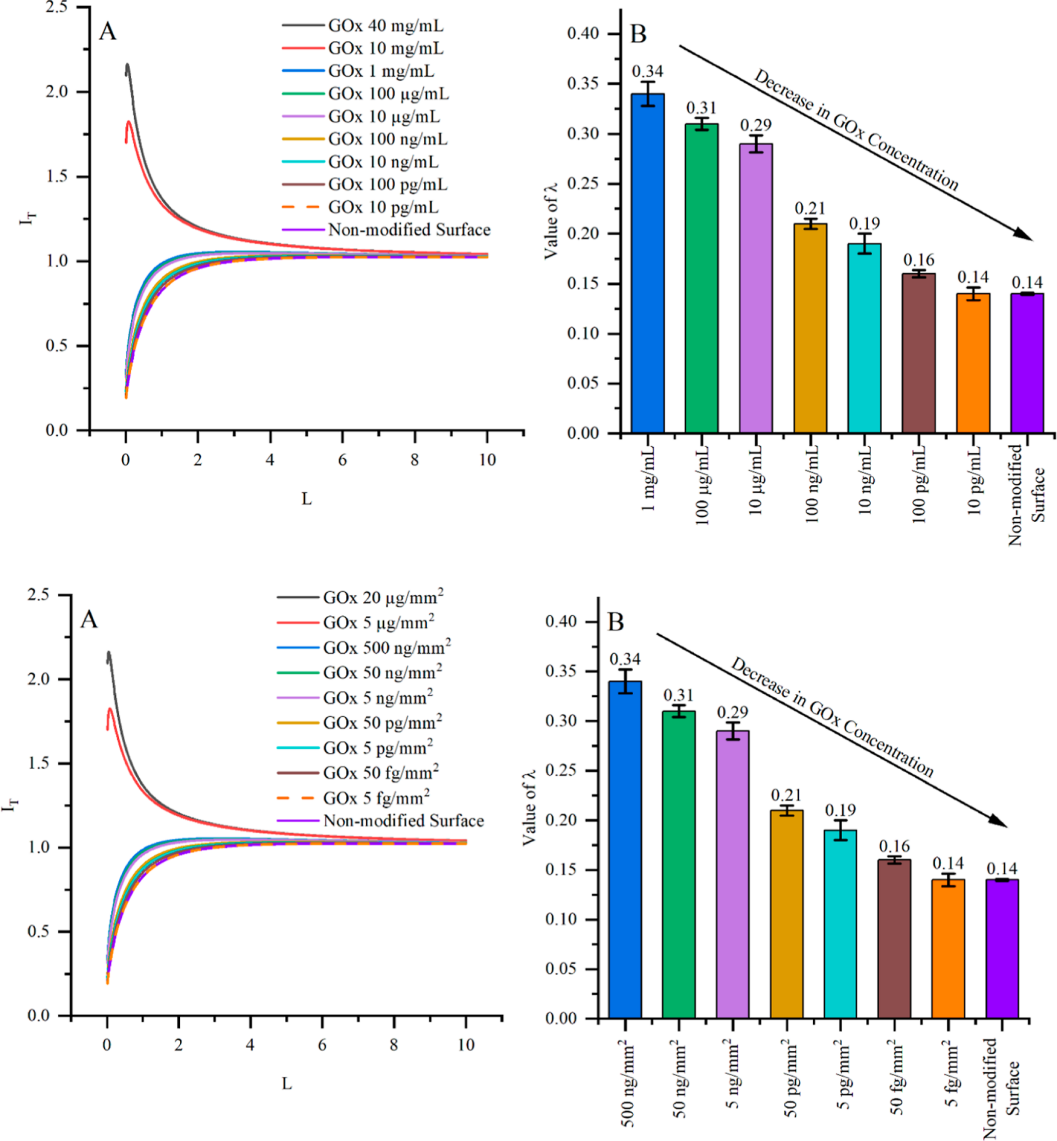
(A) FB-SECM approach curves fitted to a mathematical model, surface modified by varying GOx surface concentrations ranging from 0 to 20 μg/mm^2^. (B) Relationship between the λ kinetic parameter and GOx surface concentrations within 0 to 0.5 μg/mm^2^ range. The measurements were performed in a solution containing 120 μM of ferrocene monocarboxylic acid and 50 mM of β-d-glucose, with an applied potential of +400 mV versus Ag/AgCl.

However, when the GOx surface concentration of 50 fg/mm^2^ was assessed, then no changes in the FB-SECM-based signal were observed. This can be caused by a not sufficient catalytic activity of GOx at such low surface concentrations. Therefore, in further experiments, a much more sensitive RC-SEIM method with a [Fe­(CN)­6]^3–^/[Fe­(CN)­6]^4–^ ion (ferro/ferri)-based redox mediator system (Figure 2) was applied. At the RC-SEIM mode during the enzymatic reaction catalyzed by immobilized GOx and the reduction reaction on the UME surface, the [Fe­(CN)­6]^3–^ ion is reduced. For further research, we have assessed substrates covered by 50 pg/mm^2^ surface concentration of GOx because at this surface concentration, we have the most significant RC-SEIM signal difference compared to that determined during the assessment of the surface modified by 5 ng/mm^2^ of GOx. The other λ values determined for surfaces modified with lower than 50 pg/mm^2^ GOx concentrations were similar to that determined for a surface modified with 50 pg/mm^2^ of GOx.

**Figure 2:**
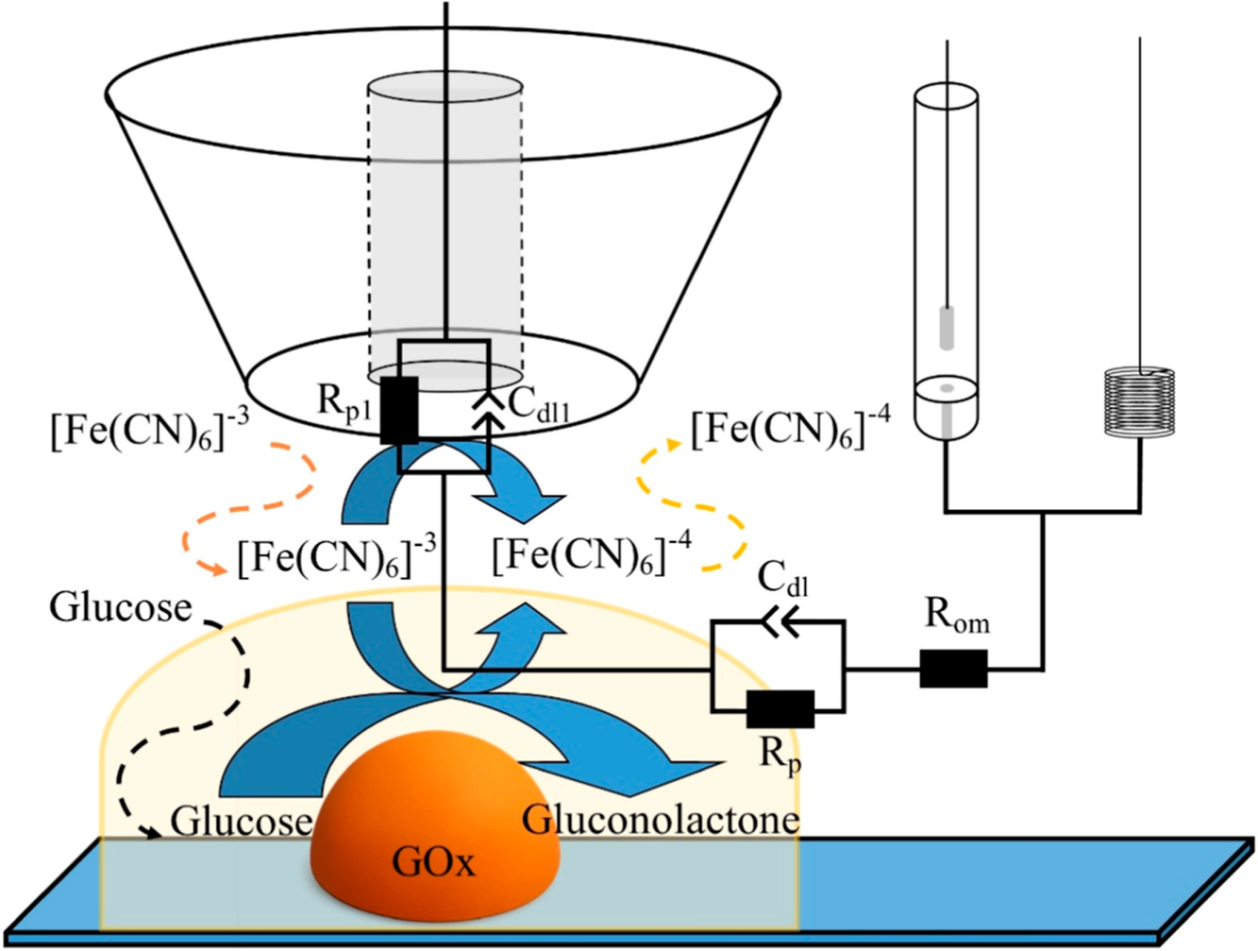
A schematic illustration of scanning electrochemical impedance microscopy measurements at the RC-SEIM mode in close proximity to glucose oxidase-modified surface in the presence of glucose.

### Experiments in the RC-SEIM Mode

Electrochemical impedance spectroscopy enables the versatile characterization of surfaces modified by various biological molecules^32^ and is well suitable for the assessment of glucose oxidase-modified surfaces.^33^ The RC-SEIM mode is suitable to measure the GOx enzymatic activity. Electrochemical impedance spectra were registered at different distances from the GOx-modified sample (not modified and GOx-modified Petri dish) surface, utilizing 1 mM ferro/ferro as a redox mediator system in the presence of 20 mM glucose. The results indicate no discernible tendencies when EIS spectra were registered close to the nonmodified Petri surface (Figure 3A), and the fitting of the data to a mathematical model revealed no significant trends in the charge-transfer resistance (Figure 3C) or the capacitance (Figure 3D). An enzyme-modified Petri dish shows differences in the impedance response; charge-transfer resistance is decreasing when UME approaches the surface (Figure 3B). Also, the enzyme-modified Petri surface (Figure 3B) exhibited distinct behavior when the distance to the surface decreased from 20 to 2 μm and the charge-transfer resistance increased by 690 MΩ (Figure 3C). The increase in the charge-transfer resistance with an decreasing tip-to-surface distance has an inverse relation between these parameters, suggesting that the immobilization of enzymes onto the surface significantly alters local electron-transfer kinetics. At the same time, no significant changes were observed in the CPE parameters (Figure 3D).

**Figure 3:**
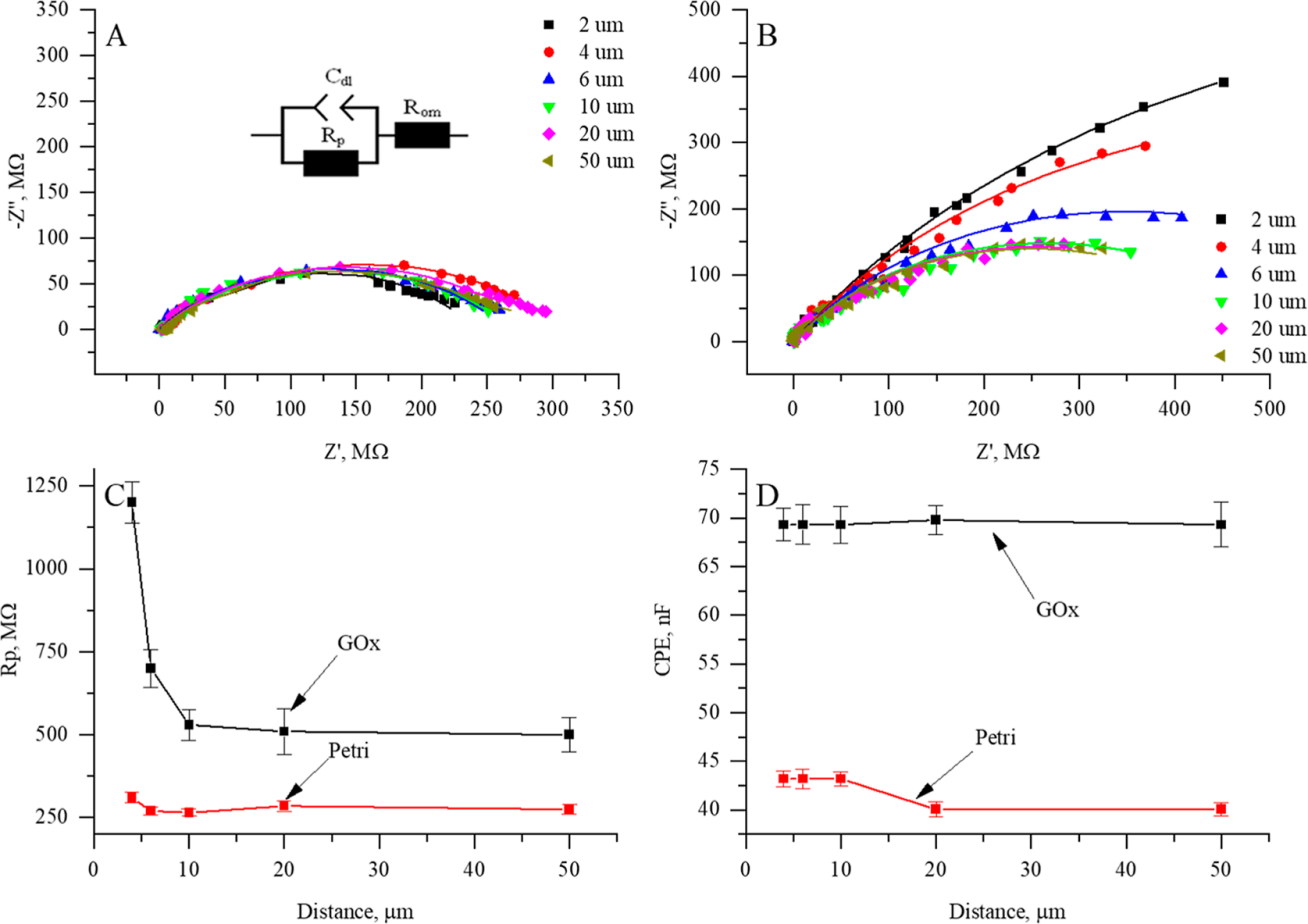
Impedance measurements were performed at different distances (2 μm, 4 μm, 6 μm, 10 μm, 20 μm, and 50 μm) UME and sample surface at a constant glucose concentration of 20 mM. (A) Nyquist plot of EIS data registered while approaching not modified Petri dish surface, (B) Nyquist plot of EIS data registered while approaching the surface modified by 50 pg/mm^2^ of GOx, (C) calculated charge-transfer resistance vs UME distance from the surface of interest, and (D) estimated double layer capacity, calculated from CPE parameters vs UME distance from the surface of interest.

Further measurements at a 4 μm distance between UME and sample surface were performed by increasing the glucose concentration in the medium from 0 to 20 mM (Figure 4). Typically, a rise in charge-transfer resistance would indicate inefficient electron transfer and imply that the enzyme is not immobilized correctly. However, as the enzyme is purposefully immobilized on the nonconductive surface, and the measurement is done in the redox-competition mode, a rise of 343 MΩ in charge-transfer resistance would indicate that the glucose oxidation reaction is facilitated. Additionally, charge-transfer resistance with respect to the glucose concentration consistently shows a low variation across repeated scans, suggesting that the system response has a high degree of reproducibility. However, testing recovery and RSD would be an important aspect of future work, especially for transitioning from proof of concept to application-ready sensors.^34–36^

**Figure 4:**
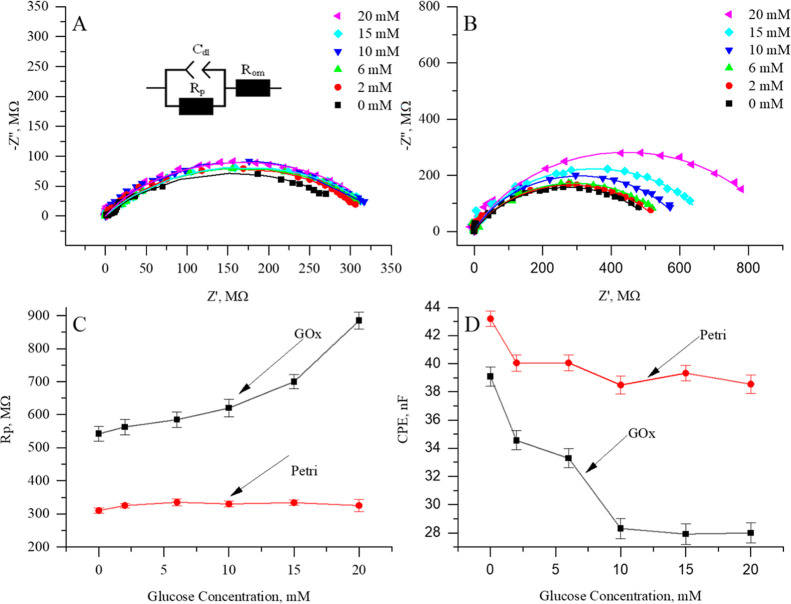
Impedance measurements were performed at varying glucose concentrations of 0 mM, 2 mM, 6 mM, 10 mM, 15 mM, and 20 mM, while the distance between UME and surface of interest remained constant of 4 μm. (A) Nyquist plot of EIS data registered while approaching the not modified Petri dish surface; (B) Nyquist plot of EIS data registered while approaching the surface modified by 50 pg/mm^2^ of GOx; (C) the calculated charge-transfer resistance as a relationship to glucose concentration; and (D) estimated double layer capacity as a relationship to glucose concentration.

Analogously to a charge-transfer resistance, a drop in the estimated capacitance by 11 nF was observed with the increase in the glucose concentration near the interface with GOx (as depicted in Figure 4C,D). While it would suggest a decrease in the effective surface area of electrodes, it could be a representative method for the assessment of enzymatic activity at GOx-modified surfaces.

At the same time, the calculated charge-transfer resistance and electrical capacitance close to the nonmodified Petri surface remained almost constant (Figure 4C,D). The slight drop in the estimated capacitance in the control experiment could occur due to the increase of solution viscosity by increasing the glucose concentration.

Although experiments were repeated 5 times, each time the Petri dish modified with GOx was replaced with a newly prepared one, as this biosensor architecture reuses only the electrodes. In the future, a fully reusable glucose biosensor platform will be assessed by a standard *t*-test to ensure the repeatability and performance^37–39^ of the developed biosensor.

Measurements at different frequencies (Figure 5) in both cases (Petri dish, Figure 5A and GOx-modified surface, Figure 5B) showed the most significant difference in normalized impedance values while registering the approach curves at 10 Hz. When measuring the electrochemical impedance spectrum at lower frequencies, several factors become more significantly pronounced: diffusion processes, double layer capacitance, electrolyte resistance, and charge-transfer resistance. The first 3 factors could contribute toward the change near the nonmodified surface (Figure 5A), and charge-transfer resistance would impact changes registered close to the modified surface. This could mean that measurement results at lower frequencies are more sensitive to the glucose oxidation reaction. However, EIS signals at lower frequencies, such as 100 mHz, are mostly affected by the noise and, due to a prolonged measurement time, cause the instability of the system. In comparison, at higher frequencies (>1 kHz), a significant reduction of sensitivity is observed due to a lower influence of charge-transfer resistance.

**Figure 5:**
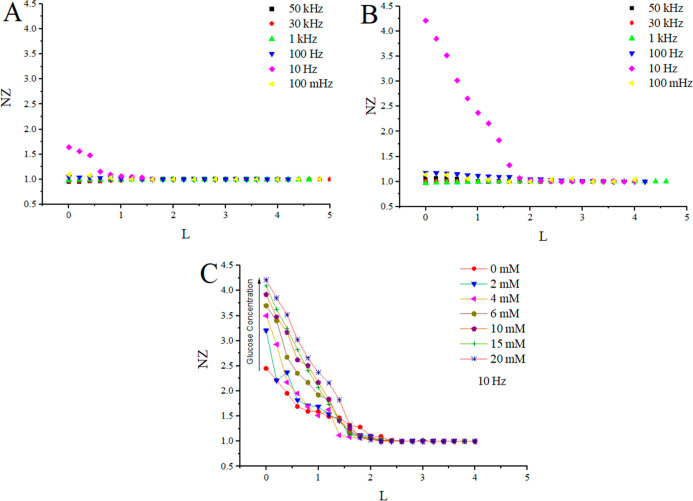
Normalized impedance was analyzed as a function of normalized distance at various frequencies at a constant glucose concentration of 20 mM. (A) Impedance registered while approaching the Petri dish at different frequencies (from 50 kHz to 100 mHz); (B) impedance data registered while approaching a surface modified by 50 pg/mm^2^ of GOx at different frequencies (from 50 kHz to 100 mHz); and (C) normalized impedance registered while approaching a surface modified by immobilized GOx (100 ng/mL) as a function of the normalized distance at different glucose concentrations in the range of 0–20 mM, measured at 10 Hz.

A noteworthy observation was made during the measurements of approach curves, especially at a frequency of 10 Hz, where the highest variation in normalized impedance values was observed, while at other frequencies significantly lower changes were observed (Table 1). Measurements conducted at different glucose concentrations (Figure 5C) revealed an increase of normalized impedance by 77%, when the glucose concentration increased from 0 to 20 mM. The initial increase of around 31% was observed between 0 and 2 mM of glucose, which is the most likely contributed by charge-transfer resistance. When the glucose concentration increases from 2 to 20 mM, an NZ increase of 1 unit of normalized impedance was observed. Furthermore, as was expected, the main changes in NZ were observed when the distance between UME and the surface of interest was lower than 2 electrode radii.

**Table 1:** ΔNZ—Differential Values Calculated in the Presence of 0 and 20 mM of Glucose at Different Frequencies

frequency applied	ΔNZ—differential values calculated in the presence of 0 and 20 mM of glucose
100 mHz	0.016
10 Hz	1.767
100 Hz	0.080
1 kHz	0.041
30 kHz	0.009
50 kHz	0.120

As the normalized impedance increases noticeably near the catalytically active surface, a 3D scan using RC-SEIM (Figure 6) produces a map of electrochemical activity. The scan in close proximity to the Petri dish surface (Figure 6A) shows no noticeable changes.

**Figure 6:**
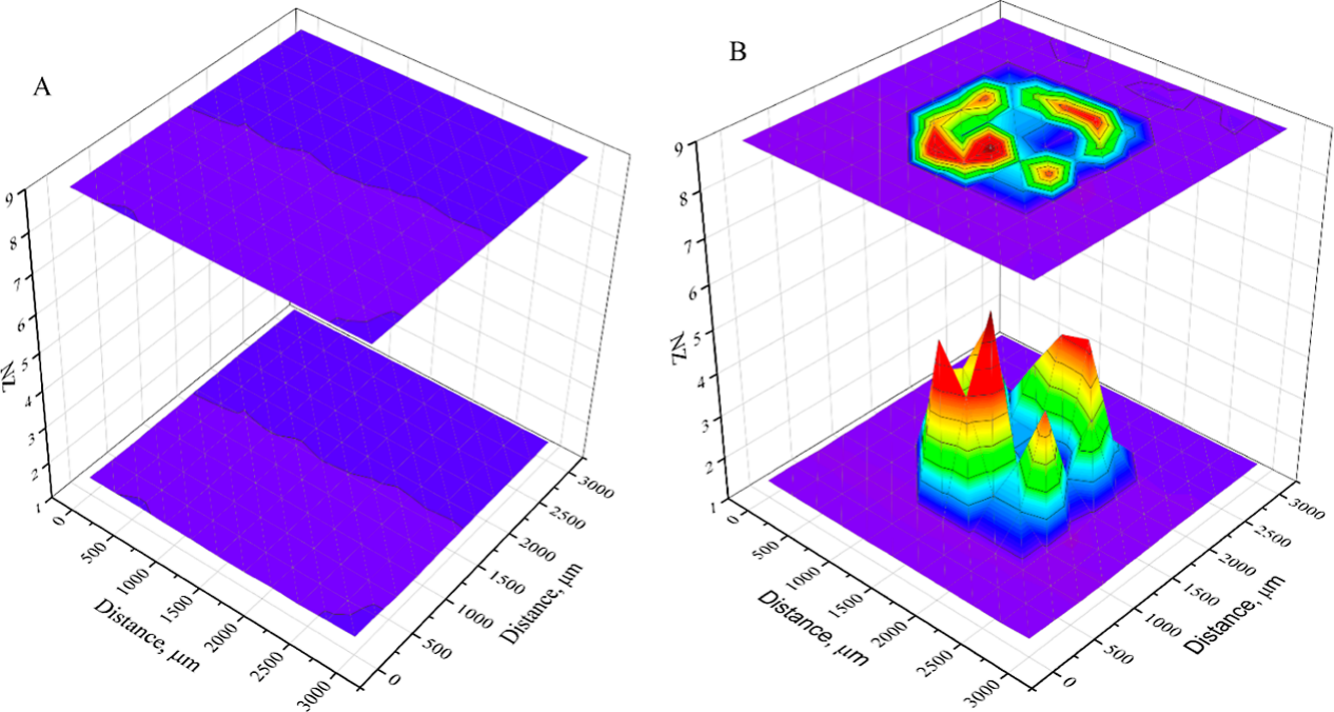
Electrochemical activity maps registered at a frequency of 10 Hz and at a constant distance of 4 μm from the sample surface: (A) electrochemical activity image registered over the not modified surface and (B) electrochemical activity image registered over the GOx-modified surface (covered by 50 fg/mm^2^ of GOx surface concentration). Measurements were performed using a step size of 200 μm, a UME scanning velocity of 200 μm/s, and a constant glucose concentration of 20 mM.

As expected, when using the glutaraldehyde-based immobilization method, most of the enzymes gathered near the edges of the drop when drying. However, the catalytic activity is unevenly spread out around the edge. This suggests that a 3D catalytic activity scan should be conducted in order to determine the optimal spot for SECM experiments. A 0.5 μL droplet of the GOx solution (100 ng/mL) was used to modify the surface. This corresponds to 50 fg of GOx per deposited spot and forms 40 fg/mm^2^ average surface density of GOx. Therefore, the estimated enzyme quantity on the active zone of the surface is on the order of tens of fentograms, and a further quantitative assessment of the enzyme should be considered. This would be one of the reasons why the highest NZ recorded at a 4 μm distance was 5.6, while the highest NZ from the approach curves was 4.2.

While the current method enables glucose detection in the 2–20 mM range, which overlaps typical physiological levels, this concentration range was selected to validate the feasibility of RC-SEIM-based glucose sensing on surfaces modified by a low surface concentration of GOx. The lowest assessed glucose concentration of 2 mM is the hypoglycemic range, which indicates that the RC-SEIM-based system is capable of detecting clinically relevant glucose concentrations. This study serves as a proof-of-concept, demonstrating that localized impedance changes can be reliably detected even at GOx-modified surfaces covered by low (50 fg/mm^2^) GOx surface concentrations while UME could be reused for a practically unlimited number of measurements. Additionally, the RC-SEIM-based system is promising for nonclinical or industrial applications, such as fermentation or food quality monitoring, where higher glucose concentrations and localized glucose sensing is required.

## Conclusions

The RC-SEIM technique has proven to be highly effective for the development of modular biosensor construction, detecting a local glucose concentration. It is suitable for the measurement of glucose concentrations ranging from 2 to 20 mM at 10 Hz AC frequencies, where the most notable changes of normalized impedance were observed. This bioanalytical system facilitates the precise quantification of glucose concentrations and also enables the detailed assessment of localized catalytic activity of the immobilized enzyme. Furthermore, the RC-SEIM technique is suitable for the assessment of the immobilized enzyme distribution. The enzyme distribution is important for the optimization of measurement conditions. Overall, RC-SEIM emerges as a robust and viable tool for the in situ performance assessment of glucose biosensors, contributing to advancements in biosensor technology and offering the potential for improved modular biosensors where the biorecognition element is immobilized on a cost-effective substrate and the ultramicroelectrode can be reused for a practically unlimited number of measurements.

## Materials and Methods

### Chemicals Used for the Investigation

A 0.1 M phosphate-acetate buffer solution (PABS) was prepared for electrochemical experiments by dissolving NaH_2_PO_4_ from Fluka Chemie GmbH, (Bucharest, Romania), Na_2_HPO_4_ from Carl Roth GmbH&Co (Karlsruhe, Germany), and CH_3_COONa from Merk (Tokyo, Japan) in deionized water. To enhance the solution’s conductivity, 0.01 M of KCl from Scharlau (Barcelona, Spain) was added. The pH of the PABS was adjusted to 6.5 with CH_3_COOH and/or NaOH both from Merk (Darmstadt, Germany).

Glucose oxidase was purchased from Merk (Saint Louis, USA), potassium ferrocyanide (K_3_[Fe­(CN)­6]^3^) and potassium ferricyanide (K_4_[Fe­(CN)­6]^4–^)—from Carl Roth GmbH&Co (Karlsruhe, Germany), ferrocene monocarboxylic acid (FcCOOH)—from Merk Darmstadt, Germany, and glucose (d-(+)-glucose (99%)) from Carl Roth GmbH&Co (Karlsruhe, Germany). Solutions of these compounds were prepared in PABS. The glucose solution was allowed to undergo mutarotation by standing overnight prior to use.

### Glucose Oxidase Immobilization

A 3 cm diameter Petri dish was cleaned using a 98.5% ethanol solution from UAB Vilniaus degtin (Vilnius, Lithuania), followed by rinsing with deionized water and drying. To form the initial glutaraldehyde layer, the cleaned Petri dish was placed in a sealed container above the 25% glutaraldehyde solution from Fluka Chemie GmbH (Buchs, Switzerland) for 15 min. Subsequently, a 0.5 μL droplet of GOx solution, with a concentration of GOx ranging from 5 fg/mm^2^ to 20 μg/mm^2^ was applied to the Petri dish’s surface and allowed to dry at ambient temperature. The sample was then exposed again to the 25% glutaraldehyde vapor for 15 min to facilitate the cross-linking of the surface-bound GOx, followed by rinsing with PABS.

### Electrochemical Cell Design

An electrochemical investigation was performed by a scanning electrochemical microscope using platinum UME with a 10 μm diameter and an Rg value of 10 purchased from Sensolytics (Bochum, Germany). A platinum wire with a surface area at least 100 times greater than that of the UME was employed as the counter electrode, while Ag/AgCl in 3 M KCl served as the reference electrode. The distance between the electrodes was maintained at 10 mm. Prior to experimentation, the UME underwent thorough electrochemical cleaning according to the manufacturer’s instructions. This procedure involved potential cycling in 0.5 M H_2_SO_4_ within a range of 0–1.2 V vs Ag/AgCl (3 M KCl), followed by rinsing with deionized water and ethanol.

### Registration of Approach Curves

The approach curves were registered in an amperometric feedback mode with a ferrocene carboxylic acid (FcCOOH) serving as a redox mediator at 120 μM concentration. The sample surface was probed using a stepwise approach of a 1 μm step with a speed of 1 μm/s and a 10 ms pause at each step. A potential of +400 mV versus Ag/AgCl (3 M KCl) was applied during the measurements. The procedure was repeated for a total of 5 iterations to ensure reproducibility.

### Calculation of Kinetics

UME parameters were determined by fitting a mathematical model^40^ to the experimentally recorded approach curves. To compare the gathered data sets, all approach curves were normalized and presented as a normalized tip current versus normalized distance according to the outline in eqs 1 and 2 ^41^

1 $$I_{T}=\frac{i_{T}}{i_{\infty }}$$

2 $$L=\frac{d}{r_{T}}$$

where *i*_T_—is the experimentally measured tip current, *r*_T_—is the UME tip radius, *d*—is the distance between the UME conducting surface and the surface of the sample, and *i*_∞_—is the steady-state current.

The steady-state current can be described according to eq 3 ^42^

3 $$i_{\infty }=4n_{e}FDCr_{T}$$

where *n*_e_ represents a number of electrons transferred in the reaction at the UME, *F* is Faraday’s constant, *D* denotes the diffusion coefficient of the target redox species, *C* is the concentration of FcCOOH, and *r*_T_ is the radius of the UME.

Glucose oxidase displays electrochemical behavior that aligns with that of neither a conventional conductor nor an insulator but rather a combination of both. As such, a mathematical model that accounts for both surface types to govern tip current can be employed^30^

4 $$I_{T}\left(L,\lambda ,R_{g}\right)=I_{T}^{cond}\left(L,R_{g}\right)+\frac{I_{T}^{ins}\left(L,R_{g}\right)-1}{\left(1+2.47{R_{g}}^{0.31}L\lambda \right)\left(1+L^{0.006R_{g}+0.113}\lambda^{-0.236R_{g}+0.91}\right)}$$

where, *I*_T_^cond^(*L*,*R*_g_) is a model for the conductive surface, *I*_T_^ins^(*L*,*R*_g_)—a model for insulating surface, λ—the kinetic constant, *R*_g_—the ratio between the insulating shroud’s radius and the conductive surface’s radius, and *L*—the normalized distance.

5 $$I_{T}^{cond}\left(L,R_{g}\right)=\alpha \left(R_{g}\right)+\frac{\pi }{4\beta \left(R_{g}\right)\mathrm{ArcTan}\left(L\right)}+\left(1-\alpha \left(R_{g}\right)-\frac{1}{2\beta \left(R_{g}\right)}\right)\frac{2}{\pi }\mathrm{ArcTan}\left(L\right)$$

6 $$\alpha \left(R_{g}\right)=\ln2+\ln2\left(1-\frac{2}{\pi }\mathrm{ArcTan}\left(\frac{1}{R_{g}}\right)\right)-\ln2\left(1-{\left(\frac{2}{\pi }\mathrm{ArcCos}\left(\frac{1}{R_{g}}\right)\right)}^{2}\right)$$

7 $$\beta \left(R_{g}\right)=1+0.639\left(1-\frac{2}{\pi }\mathrm{ArcCos}\left(\frac{1}{R_{g}}\right)\right)-0.186\left(1-{\left(\frac{2}{\pi }\mathrm{ArcCos}\left(\frac{1}{R_{g}}\right)\right)}^{2}\right)$$

8 $$I_{T}^{ins}\left(L,R_{g}\right)=\frac{\frac{2.08}{{R_{g}}^{0.358}}\left(L-\frac{0.145}{R_{g}}\right)+1.585}{\frac{2.08}{{R_{g}}^{0.358}}\left(L+0.0023R_{g}\right)+1.57+\frac{\ln R_{g}}{L}+\frac{2}{\pi R_{g}}\ln\left(1+\frac{\pi R_{g}}{2L}\right)}$$

### Measurements of Local Electrochemical Impedance

Scanning electrochemical impedance microscopy measurements were carried out over a frequency range of 100 mHz to 50 kHz, using a root-mean-square (RMS) amplitude of 10 mV and a direct current bias of +200 mV. A redox mediator consisting of 1 mM K_3_[Fe­(CN)_6_]/K_4_[Fe­(CN)_6_] was used. Measurements were performed at six different distances between the UME and the GOx-modified surface. The probe-to-sample distance was established by recording approach curves when the UME approached the plastic surface of the Petri dish. Each experiment was repeated 5 times to ensure reproducibility.

The registered data were fitted to an equivalent circuit model, which is plotted in Figure 1. The corresponding mathematical expression for the impedance is as presented

9 $$Z=\frac{Z_{Cdl}\times \left(R_{p}\right)}{Z_{Cdl}+\left(R_{p}\right)}+R_{om}$$

where *Z*_Cdl_ represents the double layer impedance (eq 10), *R*_p_ represents charge-transfer resistance, and *R*_om_ is the ohmic resistance of the solution.

The double layer impedance was modeled as a constant phase element

10 $$Z_{Cdl}=\frac{1}{Q{\left(j\omega \right)}^{\alpha }}$$

where *Q* denotes the capacitance of a constant phase element when α is equal to 1, *j* is the imaginary unit, ω represents the angular frequency, and α defines the phase angle by which the CPE impedance is shifted.

The solution resistance between the UME and the other electrodes was left uncompensated.

As demonstrated in our previous research, the distance-dependent behavior of the real and imaginary components of EIS closely resembles that observed in the FB-SECM measurements. To obtain reliable data about a specific effect or reaction, selecting an appropriate frequency that corresponds to that effect or reaction is crucial. Accordingly, AC approach curves are commonly presented as plots of normalized impedance versus normalized distance, as defined by eqs 11 and 12 ^43^

11 $$NZ=\frac{\left|Z_{d}\right|}{\left|Z_{\infty }\right|}$$

12 $$L=\frac{d}{r_{T}}$$

where |*Z*_d_| represents impedance measured at distance d from the surface, |*Z*_∞_| is the impedance measured far from the sample surface, *r*_T_ denotes the radius of the tip, and *d* is the separation between the tip and the sample surface.

### 3D Imaging by AC-SECM

A scan was performed over a 3 × 3 mm area with the UME positioned at 4 μm distance to the surface of interest. The scan used a step size of 200 μm, a scanning speed of 200 μm/s, and a 100 ms pause between steps. Measurements were conducted at a frequency of 10 Hz with a 10 mV amplitude and a +200 mV potential bias. The resulting data were normalized according to the same procedure used for AC versus distance dependence (eqs 11 and 12). This approach enabled the comparison between GOx-modified and not modified surfaces.^44^
